# Clinicians’ Assessment of Mobile Monitoring: A Comparative Study in Japan and Spain

**DOI:** 10.2196/med20.2874

**Published:** 2013-09-18

**Authors:** Shintaro Okazaki, José Alberto Castañeda, Silvia Sanz

**Affiliations:** ^1^Department of MarketingCollege of Economics & Business AdministrationUniversidad Autónoma de MadridMadridSpain; ^2^Department of MarketingCollege of Economics & Business AdministrationUniversity of GranadaGranadaSpain; ^3^Department of MarketingCollege of Economics & Business AdministrationUniversity of ValenciaValenciaSpain

**Keywords:** comparative study, health monitoring, personal innovativeness, smartphone, psychic distance

## Abstract

**Background:**

The gradual but steady shift toward telemedicine during the past decades is a clear response to important health problems that most industrialized countries have been facing. The growing elderly population and changing dietary habits have led to an increase in people with chronic diseases and overall health care expenditures. As more consumers use their mobile device as their preferred information and communication technology (ICT) device, mobile health monitoring has been receiving increasing attention in recent years.

**Objective:**

This study examines clinicians’ perception of factors determining mobile health monitoring acceptance in Japan and Spain. The study proposes a causal model consisting of innovation seeking, new ICT attributes (perceived value, time-place flexibility, and compatibility), and usage intention. In addition, cross-country differences are posited for the hypothesized relationships among the proposed constructs.

**Methods:**

A questionnaire survey was performed to test our research model and hypotheses. The sample consisted of clinicians from various medical specialties. In total, 471 and 497 usable responses were obtained in Japan and Spain, respectively.

**Results:**

In both countries, the collected data fit the model well with all the hypothesized paths among the constructs being supported. Furthermore, the moderating effects of psychic distance were observed in most of the paths.

**Conclusions:**

Our study demonstrates the importance of new ICT attributes, namely perceived value, time-place flexibility, and compatibility, in the clinicians' adoption of mobile health monitoring. In particular, our results clearly indicated that perceived medical value and ubiquitous nature of the tool are the two main benefits clinicians are likely to perceive (and appreciate) in both countries. This tendency will be stronger for those with a greater propensity to seek innovation in ICT. In terms of cross-country comparison, the strength of the path from innovation seeking to perceived value was greater in Japan than in Spain. Since the number of clinicians per 10,000 residents is substantially fewer in Japan compared with Spain, clinicians with a greater propensity to seek innovation in ICT may have perceived greater value in using mobile health monitoring to improve remote patient care.

##  Introduction

As more consumers employ information and communication technology (ICT) to manage their health and fitness, mobile health monitoring has received much attention from the health care industry [1]. Compared with other ICT tools, mobile monitoring enables clinicians more personalized and flexible control of patients’ health at a distance. One of the advantages of this monitoring system for patients is the unobtrusive, prolonged ambulatory monitoring, which allows for improved quality of life and faster response in the case of emergencies [2]. However, little attention has been paid to clinicians’ perception on this technological breakthrough. In addition, it is virtually unknown how mobile health monitoring has been accepted across borders. To fulfill this research gap, this study examines clinicians’ motivations to use mobile health monitoring in two industrialized countries, Japan and Spain.

We propose a causal model consisting of clinicians' innovation seeking, new ICT attributes (perceived value, time-place flexibility, and compatibility), and usage intention.

The model is based on Rogers’ [3] diffusion of innovation theory, mainly focusing on relative advantage and compatibility. We envisage the relative advantage of mobile health monitoring as two main factors, perceived value and time-place flexibility, while retaining compatibility as a characteristic of a new ICT that must fit not only clinicians’ work routines, but also their medical beliefs that remote control of chronic disease is beneficial [4]. These new ICT attributes turn out to be the main causes of usage intention. In addition, we posit full mediation hypotheses of new ICT attributes, since Yi et al [5] found that the impact of innovation seeking on intention to use a personal digital assistant was hardly significant in the presence of the new ICT attributes indicating full mediation. Several past studies reported similar results on the importance of individual propensity to seek innovation in directly determining user perceptions of new ICT attributes [6]. We posit that psychic distance between clinicians and patients would moderate the relationships among these constructs.

Japan and Spain were chosen for two reasons. First, both countries have developed a comprehensive public health care system that fully covers basic medical costs, with very similar medical expenditure as a percentage of gross domestic product and per capita. Second, the number of clinicians per 10,000 residents or per hospital bed is notably greater in Span than in Japan. By increasing health care costs, a lack of clinicians would drive a serious need for ICT-based health care monitoring.

## Methods

### Overview

Professional marketing research firms recruited participants in Japan and Spain. In both countries, quota sampling was applied. In an attempt to ensure a sample representative of the nation, the respondents were collected from all geographical regions, assigning a quota of clinicians per region. In Japan, 471 respondents were collected from 47 prefectures, and in Spain, 497 respondents were drawn from 17 autonomous communities. The sample consists of clinicians in diverse specialties, since the number of those specialized in diabetics is rather limited. The age ranged from 25 to 65 in Japan, and 25 to 80 in Spain.

### Statistical Analysis

#### Measurement Assessment

Before proceeding with the estimation of the structural model, we performed a full-sample confirmatory factor analysis (CFA) with six latent constructs using AMOS 19.0 [7]. Time-place flexibility was conceptualized as a second-order construct, thus time flexibility and place flexibility were added as separate first-order constructs. To take into account the recommendations by Bagozzi and Yi [8] and Bollen [9], multiple indices were used to assess the goodness of fit of the overall model: χ^2^_242_=1883.75, comparative fit index (CFI)=.93, Tucker-Lewis index (TLI)=.92, and root mean square error of approximation (RMSEA)=.084.

In a model with “good” fit, the chi-square statistic should not be significant at the 5% level. However, the literature suggests that this index becomes too sensitive in larger sample sizes [10]. The values of the CFI and TLI indices should be close to 1, although values between .90 and .95 are considered adequate [8,9]. The RMSEA index should be close to 0 [7]. Thus, all the indices, except the chi-square statistic, were in an acceptable range. In addition, all items exhibited highly standardized loadings on their intended factors. Thus, convergent validity was established.

#### Reliability and Validity

Based on the CFA results, we computed composite reliability (CR) and average variance extracted (AVE) to assess the internal consistency of the multiple measures [11]. As a benchmark, researchers generally recommend .70 and .50 as an appropriate level for the CR and AVE, respectively, in an exploratory study. All the multiple reflective constructs exceeded these criteria.

Discriminant validity is the extent to which a construct truly differs from neighboring constructs [10]. This was assessed from the latent constructs correlations matrix, where the square roots of the AVE along the diagonal are reported. The correlations between the constructs are reported in the lower left off-diagonal elements in the matrix. Fornell and Lacker [11] suggested that the average variance shared between a construct and its measures should be greater than the variance shared between the construct and other constructs in the model. Thus, discriminant validity is satisfied when the diagonal elements (square root of AVE) are greater than the off-diagonal elements in the same row and column.

#### Invariance Structure

Given our comparative purpose for the path strengths between Japan and Spain, we examined the measurement invariance across the samples, following the procedure suggested by Steenkamp and Baumgartner [12]. We tested the invariant factor loadings across the models, restricting factor loadings equally across countries. The chi-square difference between the full metric invariance model and the baseline model was significant (*P*=.008), although the other fit indices were acceptable. Thus, full metric variance was not achieved.

Yet, prior research suggests that full metric invariance is rather unrealistic and only partial invariance is required for cross-country model comparison [13]. On this basis, we next tested a series of partial measurement invariance models by sequentially relaxing the factor loadings of the items. The resulting model did not differ significantly from the baseline model (**P*=.*07). Therefore, we confirmed evidence of partial metric invariance that enabled us to assess relations in the structural model.

## Results

### Main Paths

Our structural model was examined for the full sample with the maximum likelihood method using AMOS 19.0 [7]. Most of the indices indicated an adequate model fit, except for the chi-square statistic. As explained before, the difficulty of passing this stringent test has been noted elsewhere [9]. Thus, it was judged that the multiple indices sufficiently justified the adequacy of the model’s fit to the sample data. The resulting fit indices were CFI=.93, TLI=.92, and RMSEA=.086. All the hypothesized relationships between the proposed constructs were statistically significant.

### Moderation Analysis

To test moderating effects of the country, multigroup analyses were performed using AMOS 19.0 with the maximum likelihood method. The multigroup baseline model was estimated across the two countries simultaneously, without placing any equality constraints on the hypothesized paths. Their fit indices served as initial points of comparison in addressing whether the proposed structural relationships would hold in the same way across the two groups. The chi-square value of the unconstrained or baseline model was 2572.36 (*P*<.001), with 511 degrees of freedom. In the equal path model, the path between innovation seeking and perceived value was constrained to be equal in both Spain and Japan. The difference in chi-square values between the constrained and equal path models (χ^2^_1_=3.12) suggests that the direct path between innovation seeking and perceived value was marginally greater for the Japanese sample, compared with their Spanish counterpart. This test was repeated for the path between innovation seeking and compatibility, and the one between innovation seeking and time-place flexibility. Two out of three paths were statistically greater in Japan than in Spain. As for the path between perceived value and usage intention, the difference was only marginally significant; this path was greater for the Japanese sample.

##  Discussion

### Principal Results

Our structural equation modeling results indicate that, regardless of the country, innovation seeking is a strong determinant of new ICT attributes of mobile health monitoring in terms of perceived value, time-place flexibility, and compatibility. In the comparison of the relationships among the constructs across the countries, we found that Japanese clinicians, compared with their Spanish counterparts, perceived the paths between innovation seeking and perceived value and between innovation seeking and time-place flexibility. We believe that this may be, at least partially, due to the difference in psychic distance between clinicians and patients, which is operationalized as the number of clinicians per 10,000 residents.

### Limitations

We should recognize two important limitations. First, there may be factors other than psychic distance that may have affected the cross-country differences between Japan and Spain. For example, the technology readiness may vary across countries and may have affected clinicians' perceptions on a new monitoring tool. By the same token, this study did not take into account negative factors, such as perceived risk or information security. Second, most of the respondents in both countries have not used the system before, thus their responses were based on their limited knowledge and experience.

### Conclusions

Our study serves as an initial stepping-stone in research exploring cross-country differences in clinicians’ perceptions on mobile health monitoring. Our results clearly demonstrated the importance of new ICT attributes, namely perceived value, time-place flexibility, and compatibility, in adopting mobile health monitoring in both Japan and Spain. Our study crystallized the importance of relative advantage in the framework of the Rogers’ diffusion of innovation theory [3]. Clinical value and time-place flexibility are the main benefits clinicians may perceive and appreciate from this new tool.

With regard to the cross-country comparison, the path from innovation seeking to perceived value was viewed more strongly in Japan than in Spain. This could potentially be explained by the smaller ratio of clinicians per 10,000 residents in Japan compared with Spain. Clinicians with a greater propensity to innovate ICT may have perceived greater value to use mobile health monitoring to improve remote patient care. For the same reason, the path from innovation seeking to time-place flexibility was more accentuated in Japan than in Spain, probably because Japanese clinicians are more willing to take advantage of the most important utility in mobile health monitoring—the ubiquitous nature of the device. On the other hand, there was no difference in the paths between compatibility and innovation seeking and between compatibility and usage intention.

### Future Research Suggestions

Future extension should not only overcome the limitations recognized previously, but also address additional issues directly related to mobile health monitoring adoption. For example, the concept of psychic distance between clinicians and patients has seldom been documented in prior research. Perhaps the most crucial issue here is the indicator that would represent psychic distance. The number of clinicians per bed could be a practical measure but the concept needs to be developed further. In addition to innovation seeking, there are other personal characteristics that would affect new technology adoption. For example, risk aversion, ease of use, usability, and design aesthetics, might be important factors to be considered. Furthermore, future research should examine more countries so that the obtained results can be more generalizable.

## Abbreviations

AVE: average variance extracted
CFA: confirmatory factor analysis
CFI: comparative fit index
CR: composite reliability
ICT: information and communication technology
RMSEA: root mean square error of approximation
TLI: Tucker-Lewis index
